# Membranous nephropathy concurrent with renal amyloidosis: a six-patient report and literature review

**DOI:** 10.1080/0886022X.2025.2486562

**Published:** 2025-04-22

**Authors:** Zhe Li, Jing Liu, Shuhua Zhu, Mingchao Zhang, Feng Xu, Chenfeng Jiao, Xianghua Huang, Zhen Cheng, Ke Zuo

**Affiliations:** National Clinical Research Center for Kidney Diseases, Jinling Hospital, Nanjing, China

**Keywords:** Membranous nephropathy, renal amyloidosis, renal biopsy, chronic kidney disease

## Abstract

The KDIGO 2021 guidelines suggest that individuals who test positive for serum anti-phospholipase A2 receptor (anti-PLA2R) antibodies may not require a renal biopsy to establish a diagnosis of membranous nephropathy (MN). However, it is imperative to acknowledge that MN can coexist with other chronic kidney diseases. In instances where MN is comorbid with IgA nephropathy, diabetic nephropathy, or focal segmental glomerulosclerosis, the therapeutic approach tends to be analogous. Nevertheless, there is a significant disparity in both the treatment regimen and the prognosis between MN and renal amyloidosis, with variations existing even among distinct subtypes of renal amyloidosis. Given that both MN and renal amyloidosis exhibit a predilection for the geriatric population, it is prudent to consider the possibility of MN concurrent with renal amyloidosis in elderly patients who test positive for serum anti-PLA2R antibodies. This consideration should precede a straightforward MN treatment strategy. In this report, we present six patients with MN concurrent with renal amyloidosis identified at our center over the past 14 years; in five of whom were positive for serum anti-PLA2R antibodies. We further elucidated the divergent clinicopathological characteristics and prognostic implications of these cases.

## Introduction

Membranous nephropathy (MN) and renal amyloidosis are prevalent types of chronic kidney disease (CKD) among geriatric patients. In the spectrum of elderly nephrotic syndrome (NS) etiologies reported at our center, MN was identified in 56.97% of patients, and renal amyloidosis was identified in 9.84% of patients [1]. The prevalence of these conditions has increased in recent years. A retrospective analysis of 13,519 renal biopsies performed at our center from 1979 to 2002 revealed that MN accounted for 9.89% of primary glomerular disease (PGD) and that renal amyloidosis accounted for 2.28% of secondary glomerular disease (SGD) [2]. Subsequent data from 40,759 renal biopsies conducted between 2003 and 2014 indicated an increase to 18.42% for MN in PGD and 3.63% for renal amyloidosis in SGD [3]. Historically, the diagnosis of MN and renal amyloidosis relied on renal biopsy. However, with the discovery of anti-phospholipase A2 receptor (PLA2R) antibodies as a specific serological marker for idiopathic MN [4], a positive serological result has emerged as a valid and noninvasive diagnostic alternative for MN. Consequently, in recent years, numerous patients with positive serum anti-PLA2R antibodies have declined renal biopsy in favor of initiating targeted MN therapy [5]. MN patients may exhibit comorbid IgA nephropathy, diabetic nephropathy, and focal segmental glomerulosclerosis [6], and concurrent renal amyloidosis has also been reported [7–15]. It is imperative to recognize the substantial differences in therapeutic strategies and prognoses between renal amyloidosis and other types of CKD, underscoring the necessity of meticulous review and analysis of MN patients with renal amyloidosis to distill the accumulated experience and lessons. This study reports six patients with MN concurrent with renal amyloidosis diagnosed at our center since 2011 (Tables 1 and 2), five of whom were positive for anti-PLA2R antibodies; the study also includes a literature review (Table 3).

**Table 1.** Summary of the clinical characteristics at renal biopsy and the prognoses of the 6 patients included in this study.

No.	Sex/Age (years)	Blood pressure (mmHg)	Serum anti-PLA2R antibody (RU/ml)	Urine protein (g/24h)	Alb (g/L)	Glb (g/L)	ALP (U/L)	Scr (μmol/L)	BUN (mmol/L)	NT-proBNP (pmol/L)	TnT (μg/L)	TnI (μg/L)	IVST (mm)	LVEF (%)	Serum kappa FLC (mg/L)	Serum lambda FLC (mg/L)	Serum K/λ	SIFE	Bone marrow plasma cells	Urine kappa FLC (mg/L)	Urine lambda FLC (mg/L)	Urine K/λ	Urine BJP	Treatment	Outcome
1	M/61	115/64	(+)	2.15	29.9	14.4	81	85.8	7.3	591.0*	0.019	0.06	14*	63*	35.41	178.83	0.20	(−)	3%	234.07	194.21	1.21	(−)	Discontinuation	Death
2	M/73	135/85	(+)	6.33	24.1	28.6	113	49.5	4.5	NA	NA	NA	9	70	29.41	31.35	0.94	(−)	2%	68.41	16.44	4.16	(−)	GC+TwHF	Loss to follow-up
3	M/64	137/82	667.88	10.06	26.7	19.6	70	85.8	5.1	10.3	0.007	0.03	10	62	79.76	61.39	1.30	(−)	1%	515.72	210.34	2.45	(−)	GC+CsA	CR
4	F/71	112/67	111.22	2.67	34.3	22.2	67	74.3	9.2	6.4	0.008	0.02	9	75	75.70	58.20	1.30	(−)	1%	194.00	30.90	6.28	(−)	TD	CR
5	F/53	158/98	84.22	8.43	19.0	16.5	62	144.1	7.2	25.3	0.012	0.02	9	60	68.70	84.90	0.81	(−)	2%	622.50	151.00	4.12	(−)	Thalidomide	Compromised renal function
6	M/57	155/76	(−)	8.66	30.4	16.8	71	84.9	9.1	317.9	0.068	0.16	16	61	37.20	69.60	0.53	(−)	1%	52.50	115.50	0.50	(−)	BD+ASCT	PR

*Results 18 months after renal biopsy.

PLA2R, phospholipase A2 receptor; Alb, albumin; Glb, globulin; ALP, alkaline phosphatase; Scr, serum creatinine; BUN, blood urea nitrogen; NT-proBNP, N-terminal pro-B-type natriuretic peptide; TnT, Troponin T; TnI, Troponin I; IVST, interventricular septum thickness; LVEF, left ventricular ejection fraction; FLC, free monoclonal light chains; κ/λ, kappa/lambda; SIFE, serum immunofixation electrophoresis; BJP, bence jones protein; GC, glucocorticoids; TwHF, tripterygium wilfordii Hook F; TD, Thalidomide + Dexamethasone; BD, Bortezomib + Dexamethasone; ASCT, autologous stem cell transplantation; CR, complete remission; PR, partial remission; NA, not available.

**Table 2.** Summary of the renal pathological features of the 6 patients included in this study.

No.	Type of amyloidosis	Congo red staining (glomeruli / renal interstitium /skin fat / rectal mucosal)	IFA with anti-K/λ chains antibodies (glomeruli / renal interstitium /skin fat / rectal mucosal)	Routine IFA with glomeruli									
				IgA	IgM	C3	C1q	IgG	IgG1	IgG2	IgG3	IgG4	PLA2R
1	AL	(−) / (+) / (+) / NA	(κ+, λ+) / (κ-, λ-) / (κ-, λ+) / (κ-, λ+)	(−)	(−)	2+	(−)	2+	2+	(−)	1+	2+	NA
2	Undefined	(−) / (+) / (+) / NA	(κ+, λ+) / (κ-, λ-) / NA / NA	(−)	(−)	3+	(−)	2+	2+	2+	1+	3+	NA
3	ApoA-I	(+) / (+) / (−) / (−)	(κ+, λ+) / (κ-, λ-) / (κ-, λ-) / (κ-, λ-)	(−)	1+	2+	1+	2+	2+	(−)	2+	2+	(+)
4	AL	(+) / (−) / (+) / NA	(κ+, λ+) / (κ-, λ-) / (κ-, λ+) / NA	(−)	(−)	2+	(−)	2+	2+	1+	1+	2+	(+)
5	Finnish	(+) / (+) / NA / NA	(κ+, λ+) / (κ-, λ-) / NA / NA	1+	1+	2+	1+	2+	2+	1+	2+	3+	(+)
6	AL	(+) / (+) / (−) / (+)	(κ+, λ+) / (κ-, λ-) / (κ-, λ-) / (κ-, λ-)	(−)	1+	2+	1+	2+	2+	(−)	1+	1+	(−)

IFA, immunofluorescence assay; κ/λ, kappa/lambda; PLA2R, phospholipase A2 receptor; AL, amyloid light-chain; ApoA-I, Apolipoprotein A-I; NA, not available.

**Table 3.** Literature reports on membranous nephropathy with amyloidosis.

References	Nation	Sex/Age (years)	Type of amyloidosis	Serum anti-PLA2R antibody (RU/ml)	Glomerular PLA2R deposition	Blood pressure (mmHg)	Urine protein (g/24h)	Alb (g/L)	Scr (umol/L)	Treatment	Outcome
Bohel et al. [7]	Germany	F/57	Undefined (Related to RA)	NA	NA	120/70	NA	NA	53	NA	NA
		M/52				120/80			141.4		
		M/59				130/65			123.8		
Muso et al. [8]	Japan	M/61	Undefined (Related to WM)	NA	NA	NA	3.9	33.2	Normal	GC+CTX	Death
Kuroda et al. [9]	Japan	6 cases	AA (Related to RA)	NA	NA	NA	NA	NA	NA	NSAIDs + DMARD	NA
Li et al. [10]	China	F/74	ALECT2	278.6	(+)	Hypertension	11.26	26.5	118	Support therapy	Stable renal function
		F/61		(−)	(+)	Normal	2.44	22.3	76	Support therapy	
		M/79		143	(+)	Hypertension	7.92	24.7	541.8	GC + CTX	
		F/61		(−)	(+)	Hypertension	3.89	31.4	70.2	Chelation therapy	
Morel et al. [11]	France	M/48	AL (Related to WM)	THSD7A(+)	THSD7A(+)	NA	NA	23	73.4	ASCT+RTX	CR
Wang et al. [12]	China	M/39	AL	(−)	(+)	99/68	8.01	13.1	95.3	Support therapy	NA
Zhang et al. [13]	China	M/48	AL	(+)	NA	146/96	17.84	18.3	97.3	RTX+GC+CTX	CR
Xu et al. [14]	China	M/60	ALECT2	(+)	(+)	114/69	12.5	15	78.68	GC+CsA	CR
Shaheen et al. [15]	America	M/48	ALECT2	(−)	(−)	NA	4.2	15	167.96	NA	NA

PLA2R, phospholipase A2 receptor; Alb, albumin; Scr, serum creatinine; RA, rheumatoid arthritis; WM, Waldenstrom macroglobulinemia; GC, glucocorticoids; CTX, cyclophosphamide; AA, Amyloid A; NSAIDs, nonsteroidal antiinflammatory drugs; DMARD, disease modifying antirheumatic drugs; ALECT2, leukocyte chemotactic factor 2; ASCT, autologous stem cell transplantation; RTX, rituximab; CsA, cyclosporine A; CR, complete remission; NA, not available.

## Case 1

**Figure 1.**
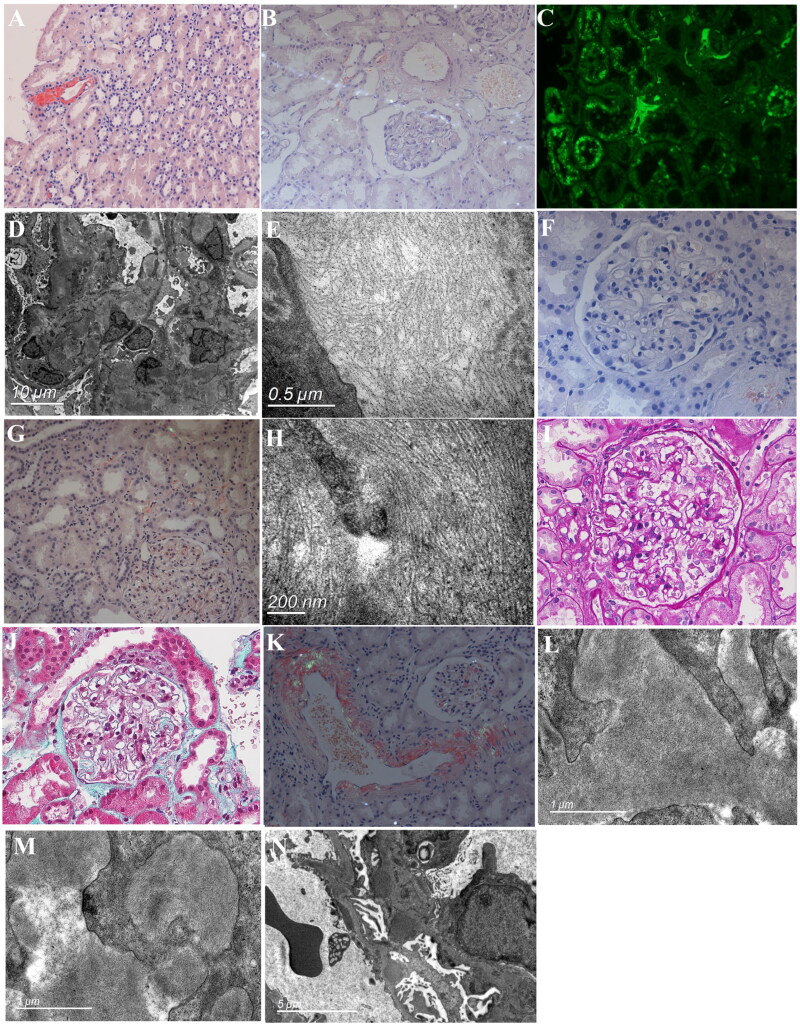
Renal pathology of the patients in this series. A. Congo red staining positivity in renal interstitial arterioles (200×). B. Congo red positivity in the renal interstitium with glomerular negativity (200×). C. IFA shows Apo A-I positivity in the renal interstitium. D. EM reveals amorphous, low-electron-density deposits in the mesangial and paramesangial areas, subendothelium, and arterial walls. E. EM displays non-branching fibrous filaments measuring 8 to 13 nm in diameter. F. Congo red staining positivity in the glomerular mesangium and vascular loops (400×). G. Congo red positivity in the glomeruli and interstitium (200×). H. EM shows a disorderly arrangement of nonbranching filaments 7 to 11 nm in diameter in the mesangial region with a parallel fascicular and whorled pattern. I. PAS staining shows weakly positive material deposition in glomeruli and renal interstitial vessels (400×). J. Masson staining shows a significant amount of eosinophilic material deposition on the epithelial side of the glomeruli, appearing bright green (400×). K. Positive Congo red staining in glomeruli and renal interstitial vessels (200×). L&M. EM reveals disordered, nonbranching fibrillary structures with a diameter of 8 to 12 nm in the mesangial area, glomerular basement membrane epithelial side, and interstitial artery walls. N. EM shows densely distributed electron-dense deposits on the epithelial side of the glomerular basement membrane, with extensive fusion of the podocyte foot processes.

A 61-year-old male patient was admitted to our center in August 2011, presenting with a one-year history of peripheral and scrotal edema. The laboratory findings at admission are presented in Table 1. A renal biopsy was conducted, leading to a diagnosis of MN. At that time, an immunofluorescence assay (IFA) of renal tissue demonstrated positive results for both kappa (κ) and lambda (λ) light chains, while Congo red staining was not conducted. The patient was subsequently placed on a regimen of 24 mg/day methylprednisolone and 2 mg/day tacrolimus. This intervention resulted in a decrease in urinary protein to 1.43 g/24 h and an increase in Alb to 34.0 g/L. In June 2012, he had pemphigus, prompting an adjustment in therapy. Methylprednisolone was decreased, and *Tripterygium wilfordii* Hook F (TwHF) [16,17] was introduced, while tacrolimus was discontinued. This change was associated with an increase in urinary protein to 3.85 g/24 h and a decrease in Alb to 27 g/L. Concurrently, he experienced exacerbation of edema; oliguria; and recurrent diarrhea, characterized by 6 to 8 loose stools per day. From March 2013, he experienced orthostatic dizziness, corresponding to a blood pressure of 90/60 mmHg. The edema intensified, and despite continuous diuretics, the patient’s urine output decreased to 300 mL/day. He also complained of profound fatigue, chest tightness and nocturnal dyspnea. After admission, the patient experienced an episode of syncope upon ambulation, with blood pressure dropping to 75/50 mmHg. The serum free light chain kappa/lambda (κ/λ) ratio was 0.20. Subsequent skin and fat biopsies, along with Congo red staining, returned positive results. IFA of the skin tissue and rectal mucosa was positive for λ light chain and negative for κ light chain. Examination of previous renal tissue with Congo red staining demonstrated positivity in a few arteriolar wall segments within the renal interstitium, with the glomeruli remaining negative (Figure 1A). On the basis of these findings, the patient was diagnosed with immunoglobulin amyloid light-chain (AL) renal amyloidosis. Ultimately, the patient opted to discontinue treatment, was discharged, and succumbed to cardiac failure two months after discharge.

## Case 2

A 73-year-old male patient presented in September 2012 with chief complaints of edema, proteinuria and intermittent diarrhea for eight months. Prior treatment at other medical centers had included albumin infusion, furosemide, and herbal formulations (specific components unknown), yet the conditions relapsed. The laboratory findings at admission are presented in Table 1. A renal biopsy was conducted, revealing findings consistent with MN. Furthermore, Congo red staining of the renal tissue and skin was positive in discrete areas of the renal interstitium and adipose tissue but negative within the glomeruli (Figure 1B). Skin tissue staining for lysozyme was negative (Supplemental Figure 1). The patient declined a rectal mucosa biopsy. Owing to the focal deposition of amyloid, insufficient material was obtained for a definitive classification of amyloidosis. Consequently, a diagnosis of MN concurrent with renal amyloidosis was established. The patient was treated with prednisone 30 mg/day and TwHF 60 mg/day but was lost to follow-up after discharge.

## Case 3

A 64-year-old male patient with a 5-month history of edema and proteinuria was admitted in November 2016. The laboratory findings at admission are presented in Table 1. Renal biopsy was consistent with MN. In addition, periodic acid–Schiff (PAS) and hematoxylin and eosin (HE) staining revealed homogenously pale staining deposits in the mesangial areas, peripheral loops, renal interstitium, and interlobular artery walls. Congo red staining was positive in the glomeruli, interstitium, and interstitial vessels. IFA revealed apolipoprotein A-I (ApoA-I) positivity in the renal interstitium but not in the glomeruli (Figure 1C). Electron microscopy (EM) revealed amorphous, low-electron-density material in the mesangial and paramesangial areas, subendothelium, and arterial walls (Figure 1D), with high-power examination revealing nonbranching fibrous filaments 8 to 13 nm in diameter (Figure 1E). Genetic screening for Apo A-I mutations was negative in the patient and his offspring. The patient was finally diagnosed with MN concurrent with nonhereditary Apo A-I renal amyloidosis. Treatment commenced with prednisone 30 mg/day and cyclosporine 100 mg/day, resulting in a decrease in urinary protein and an increase in Alb. Prednisone was tapered off by December 2017, cyclosporine was discontinued in April 2019, and maintenance therapy with TwHF was initiated. As of July 2024, the patient’s urinary protein had decreased to 0.26 g/24 h, Alb had increased to 48.1 g/L, Scr had increased to 101.7 μmol/L, and serum anti-PLA2R antibody was undetectable.

## Case 4

A 71-year-old female patient was admitted in January 2019 and presented with lower limb edema and proteinuria for 20 days. The laboratory findings at admission are presented in Table 1. The renal biopsy was consistent with MN, with additional weakly PAS-positive material distributed in the mesangial areas and vascular loops, and Congo red staining was positive in both renal (Figure 1F) and skin adipose tissues. IFA of the skin adipose tissue revealed positivity for λ light chain and negativity for κ light chain. The patient was diagnosed with MN concurrent with AL renal amyloidosis. She was treated with the TD (thalidomide + dexamethasone) regimen, consisting of thalidomide 50 mg/day and dexamethasone 10 mg weekly, for a period of 2 years. Follow-up continued through December 2023, at which time evaluation indicated that the urinary protein level had decreased to 0.12 g/24 h, the Scr level was 71.6 μmol/L, the Alb level was 47.1 g/L, and the serum free light chain κ/λ ratio was 1.07.

## Case 5

A 53-year-old female patient presented in May 2019 with a two-year history of proteinuria and elevated Scr. The laboratory findings at admission are presented in Table 1. A renal biopsy was performed, confirming MN. Additionally, histological examination revealed weakly positive material in the glomeruli, interstitium, and vascular walls *via* light microscopy with HE staining and weak PAS positivity. Congo red staining was positive in the glomeruli and renal interstitium (Figure 1G). EM revealed a disorderly arrangement of nonbranching filaments with a diameter of 7–11 nm in the mesangial region, with a parallel fascicular and whorled pattern (Figure 1H). Whole-exome sequencing revealed a heterozygous mutation, c.640G > A (p.D214N aspartic acid → asparagine), in exon 4 of the GSN gene (Supplemental Figure 2). The patient was diagnosed with MN concurrent with hereditary Finnish renal amyloidosis. Treatment was initiated with prednisone 30 mg/day, tacrolimus, and losartan. After one month, the urinary protein level had further increased to 12.6 g/24 h, the Alb level was 25.7 g/L, the Scr level was 143.2 μmol/L, and the anti-PLA2R antibody level had decreased to 60.81 RU/ml. Owing to the side effects of palpitations and hand tremors, prednisone and tacrolimus were discontinued. She was treated with thalidomide 50 mg/day for 14 months, with subsequent levels of urinary protein at 4.84 g/24 h, Alb at 29.2 g/L, Scr at 306.8 μmol/L, and anti-PLA2R antibody at 1.99 RU/mL. Thalidomide was discontinued, and the patient was transitioned to supportive care. As of the last follow-up in September 2024, the patient remained alive and did not require dialysis.

## Case 6

A 57-year-old male patient presented in July 2022 with a two-month history of bilateral lower limb edema and proteinuria. The laboratory findings at admission are presented in Table 1. Renal biopsy revealed features of glomeruli and renal interstitial vessels with faint HE staining and weakly PAS-positive material deposition (Figure 1I). There was a significant amount of eosinophilic material deposited on the epithelial side of the glomeruli, which appeared bright green upon Masson staining. (Figure 1J). The peripheral loops showed ‘spiky’ argentaffin deposits. Congo red staining was positive in the glomeruli, renal interstitial vessels and rectal mucosa (Figure 1K). Under EM, disordered, nonbranching fibrillary structures with a diameter of 8–12 nm were observed in the mesangial area, the epithelial side of the glomerular basement membrane, and the interstitial artery walls (Figure 1L and M). Densely distributed electron-dense deposits were observed on the epithelial side of the glomerular basement membrane, with extensive fusion of the podocyte foot processes (Figure 1N). Laser microdissection and mass spectrometry (LMD-MS) proteomic analysis of glomerular amyloid deposits revealed the predominant presence of immunoglobulin lambda (Ig λ) and a significant abundance of the amyloid chaperone Apolipoprotein A-IV (Supplemental Figure 3). The patient was diagnosed with MN concurrent with AL renal amyloidosis. He was initially treated with the TD regimen (thalidomide 50 mg/day and dexamethasone 20 mg weekly) for two weeks but had to discontinue due to drowsiness, fatigue, and dizziness. The BD (bortezomib + dexamethasone) regimen was subsequently initiated (bortezomib 1.3 mg/m^2 plus dexamethasone 40 mg on Days 1, 4, 8, and 11) for three cycles. Six months after treatment, his Scr level was 99.89 μmol/L, his urine protein level was 2.25 g/24 h, his Alb level was 38.2 g/L, and his serum free light chain κ/λ ratio was 0.26. He underwent autologous stem cell transplantation (ASCT) in April 2023. Follow-up in September 2023 showed that his Scr level was 94.6 μmol/L, his urine protein level was 1.67 g/24 h, his Alb level was 39.9 g/L, and his serum free light chain κ/λ ratio was 0.82.

## Discussion

The clinical and pathological features of patients with MN and renal amyloidosis diverge from the traditional conception of renal amyloidosis in isolation. In 1978, Bohle et al. initially documented three cases of MN concurrent with renal amyloidosis. Given the diagnostic constraints of the era, the precise classification of renal amyloidosis remained elusive, with an assumed association with rheumatoid arthritis [7]. Advancing renal pathological techniques, coupled with the widespread adoption of LMD-MS and genetic testing, have facilitated the diagnosis of an increasing number of cases over the past decade [18]. In this study, we aggregated and analyzed six cases from our series and nineteen from the extant literature. Among the renal amyloidosis subtypes in MN patients, the AL, amyloid A (AA), and leukocyte chemotactic factor 2 (ALECT 2) types were each represented in six cases. Nonetheless, in contrast to the 456 and 474 cases of renal amyloidosis previously reported by our center [19] and the Mayo Clinic [20], where the AL type predominated at 93.0% and 85.9%, respectively, our data suggest that while the AL type is a significant subtype in MN concurrent with renal amyloidosis, it is not exclusively predominant. This suggests that the coexistence of the two diseases is not a single entity but rather a coincidental association. In this study, five out of six patients tested positive for serum anti-PLA2R antibodies. Although previous studies have reported that approximately 75% of MN patients are positive for serum anti-PLA2R antibodies, considering that amyloid has a dual role in the immune system—both promoting inflammatory responses and potentially regulating immunity and maintaining immune tolerance—whether it contributes to immune-mediated MN requires further exploration [5,21]. Within our series, 30.2% of patients with AL renal amyloidosis presented with hypotension, 67.9% presented with positive serum immunofixation electrophoresis (SIFE), 46.7% presented with cardiac involvement, and 12.6% presented with hepatic involvement [22]. At another Chinese center, serum-free monoclonal light chains (SFLCs) were identified in 15.1% of patients with AL renal amyloidosis. Glomerular amyloidosis was a universal finding, with renal interstitial amyloidosis detected in 45.2% of patients [23]. In our study, none of the six patients with MN and renal amyloidosis exhibited hypotension. SIFE was uniformly negative. No instances of cardiac or hepatic involvement were observed, and no patient had detectable SFLC. These findings highlight the atypical clinical presentation and low positivity rate of laboratory tests in these amyloidosis patients, emphasizing the critical role of renal biopsy in achieving a definitive diagnosis.

MN concurrent with renal amyloidosis can be easily overlooked and misdiagnosed. MN can obscure the clinical and pathological characteristics of renal amyloidosis. In our series, NS emerged early in the course of MN, leading to renal biopsy at an earlier stage than in patients with isolated renal amyloidosis. The classic clinical features of renal amyloidosis, such as hypotension, diarrhea, heart failure, and hepatomegaly, along with abnormal laboratory findings, including SIFE, SFLC, and abnormal bone marrow plasma cells, were not yet apparent. Commercial antibodies typically target the constant region of light chains, and some amyloid proteins derived from the variable light chain region may not be recognized by these antibodies, complicating the diagnostic process [24]. Clinicians may be misled by a positive serum anti-PLA2R antibody result to forgo requesting Congo red staining of renal tissue. Pathologists’ inherent experience might lead to a focus on glomerular deposits of homogenous, pale material under HE and PAS staining when screening for renal amyloidosis. However, in our study, amyloid deposition was more prevalent in the renal interstitium than in the glomeruli, and amyloidosis was confirmed by biopsies of other sites in some patients. We have previously reported that the sensitivity of skin plus subcutaneous fat and rectal mucosal biopsy for diagnosing AL renal amyloidosis is 98.9%. If both biopsies are negative, the likelihood of a missed diagnosis is reduced [25]. Therefore, in elderly patients with positive serum anti-PLA2R antibodies who do not undergo renal biopsy, consideration should be given to skin fat and rectal mucosa biopsy to assess the risk of MN complicated by renal amyloidosis. Renal amyloidosis, particularly AL renal amyloidosis, can induce glomerular basement membrane spicules that may closely resemble the pathological features of MN [20]. Additionally, congophilic fibrillary glomerulonephritis (FGN) can present with features resembling both MN and renal amyloidosis [26]. These conditions should be carefully considered in the differential diagnosis of concurrent MN and renal amyloidosis. Therefore, a comprehensive histopathological evaluation, including DNAJB9 immunohistochemistry, EM, immunofluorescence, and MS, is crucial for achieving an accurate diagnosis.

Determining the type of amyloid fibril in patients with renal amyloidosis is essential for guiding treatment. The current diagnostic gold standard involves examination of biopsy tissue stained with Congo red, which exhibits characteristic green birefringence under cross-polarized light [27]. While immunohistochemistry remains the predominant method for classifying amyloid fibrils, LMD/MS has demonstrated superior diagnostic accuracy [28]. Genetic sequencing is mandatory when hereditary amyloidosis is suspected [29]. In the early years at our center, the diagnosis of renal amyloidosis was missed in the patient in Case 1 and reconsidered following the appearance of classic clinical symptoms, and the patient in Case 2 also had an undetermined type of renal amyloidosis. However, with the ongoing enhancement of clinical awareness and diagnostic techniques, our capacity for accurate diagnosis has significantly improved in recent years.

There is no universally accepted protocol for the treatment of MN patients who also have renal amyloidosis. In this study, the patient in Case 1 was initially treated for MN, resulting in a decrease in proteinuria and an increase in Alb, but this also obscured the progression of AL renal amyloidosis. It is imperative to initiate therapy for AL renal amyloidosis before the onset of irreversible involvement of vital organs. The patient in Case 4 in this study was treated with the TD regimen immediately upon diagnosis, achieving complete remission (CR) without the need for additional MN-specific medications. In contrast, the patient in Case 6 exhibited no hematologic response and only a partial renal response following BD regimen treatment, ultimately requiring ASCT to achieve CR. Two other patients described in the literature were treated with ASCT in combination with rituximab (RTX) and RTX in combination with glucocorticoids (GCs) or cyclophosphamide (CTX), and both achieved CR [11,13]. Thus, early diagnosis of MN concurrent with AL renal amyloidosis should prioritize treatment of the latter, with the potential for CR. Apo A-I renal amyloidosis predominantly affects the renal interstitium, has a chronic course, and is relatively indolent. In 38% of patients, renal disease progresses slowly, and only 13% of patients progress to dialysis [30]. Moreover, organ transplant patients exhibit very high graft survival rates [31]. However, targeted treatment options are scarce. In this study, the patient in Case 3 achieved CR with targeted MN therapy. Finnish amyloidosis is rare, and although there is no specific pharmacological treatment currently available, the average life expectancy is similar to that of the general population [32]. A case of end-stage Finnish renal amyloidosis was reported, in which the transplanted kidney functioned stably, and urinary tests remained negative six years after transplantation [33]. In this study, the patient in Case 5 was treated consecutively for MN and renal amyloidosis. Despite serum anti-PLA2R antibody becoming undetectable, she continued to experience significant proteinuria and deterioration of renal function. The lack of therapeutic response is believed to be associated with the impairment of renal function at the initial treatment stage; however, the disease progression is slow and has not yet necessitated dialysis. Other studies have reported the treatment of MN concurrent with ALECT2 renal amyloidosis, with favorable outcomes achieved using supportive care, chelation therapy, GC in combination with CTX, and GC in combination with cyclosporine A [10].

In conclusion, our study presents six patients with MN concurrent with renal amyloidosis, underscoring the necessity of enhancing vigilance for the comorbidity of MN with other CKD, notably renal amyloidosis. All such patients should ideally undergo renal biopsy. Even if serum anti-PLA2R antibodies are positive, Congo red staining should be routinely performed, and if feasible, EM should be conducted. Furthermore, the therapeutic approach for these patients should be individualized on the basis of the specific subtypes of renal amyloidosis.

## Supplementary Material

Supplemental Material

Supplemental Material

Supplemental Material

## Data Availability

All the data supporting our findings is contained within the manuscript.
