# Supplementary figures and images for: Membranous nephropathy concurrent with renal amyloidosis: a six-patient report and literature review
Source: Ren Fail. 2025 Apr 22;47(1):2486562. doi: 10.1080/0886022X.2025.2486562 (PMC12016244; doi:10.1080/0886022X.2025.2486562)

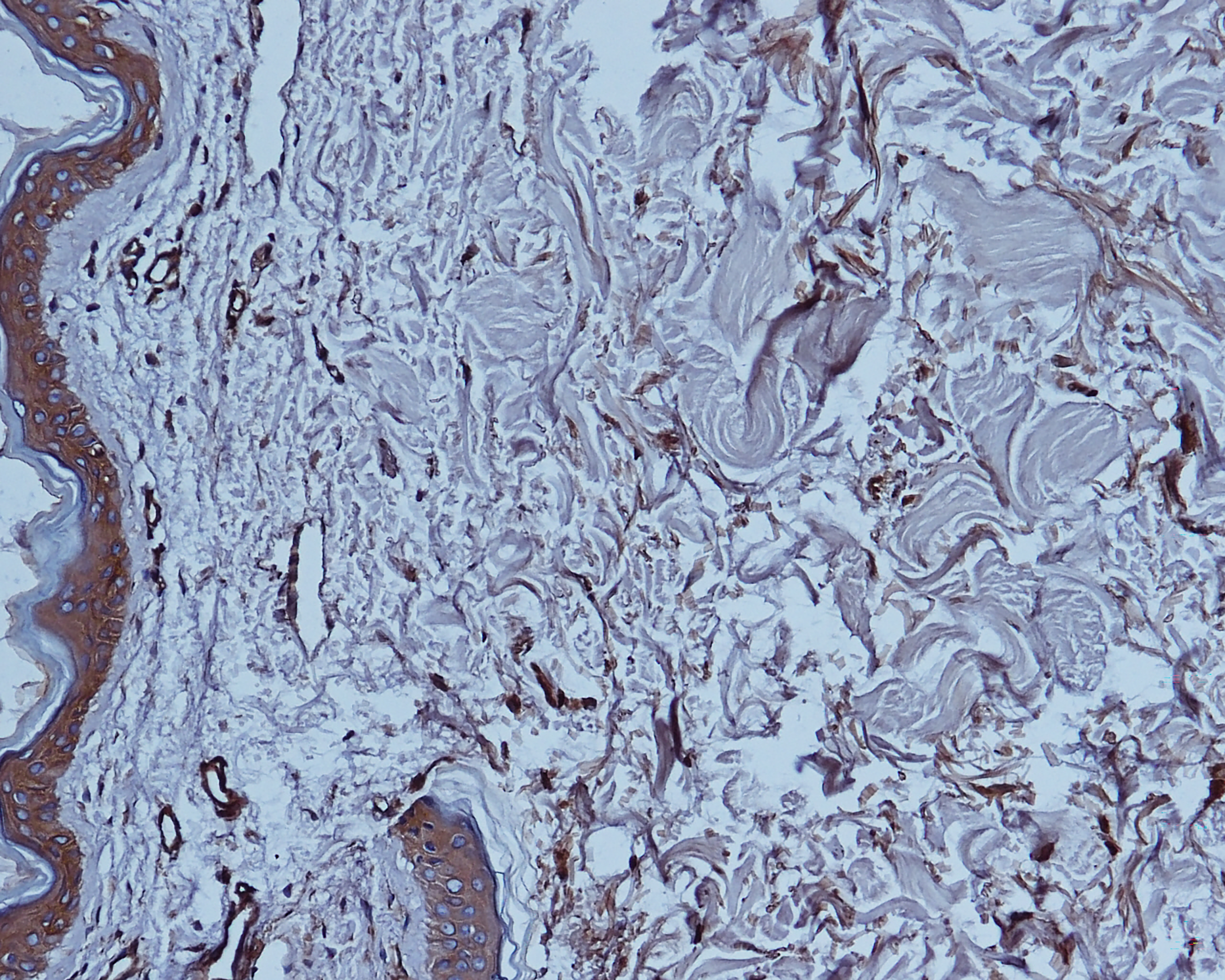

Supplement: Supplemental Material [file IRNF_A_2486562_SM2286.tif]

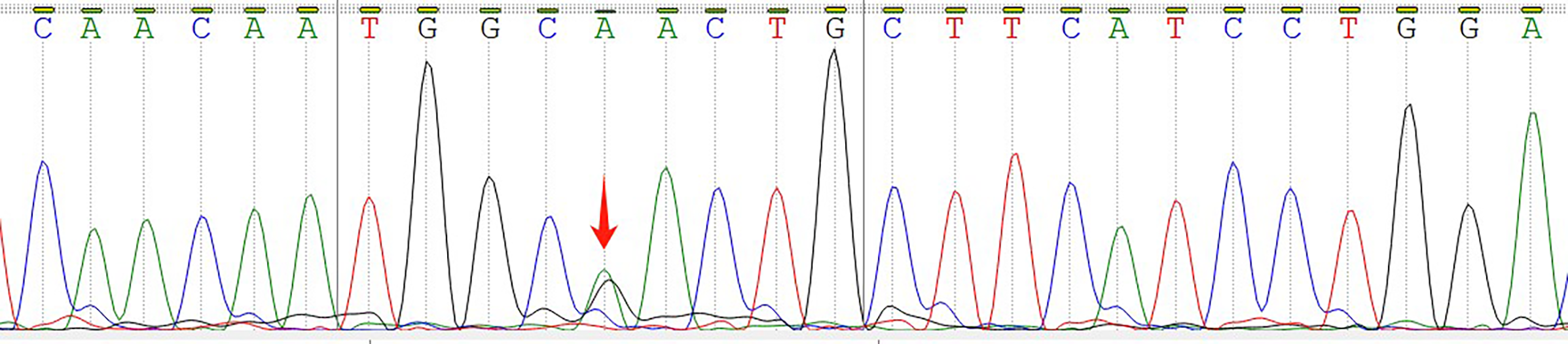

Supplement: Supplemental Material [file IRNF_A_2486562_SM2281.tif]

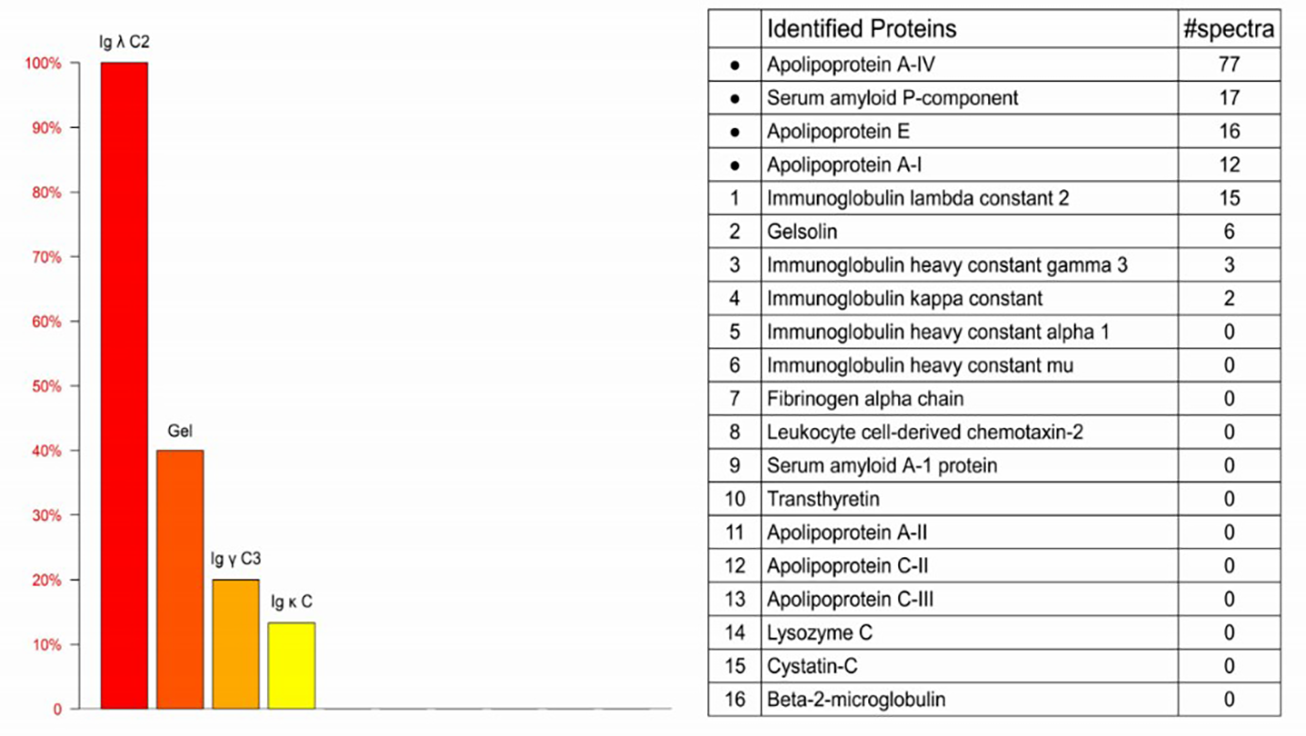

Supplement: Supplemental Material [file IRNF_A_2486562_SM2277.tif]
